# WGCNA analysis of the effect of exogenous BR on leaf angle of maize mutant *lpa1*

**DOI:** 10.1038/s41598-024-55835-7

**Published:** 2024-03-04

**Authors:** Xiangzhuo Ji, Qiaohong Gao, Zelong Zhuang, Fangguo Chang, Yunling Peng

**Affiliations:** 1 State Key Laboratory of Aridland Crop Science, Gansu Agricultural University, Lanzhou, 730070 China; 2 College of Agronomy, Gansu Agricultural University, Lanzhou, 730070 China; 3 Gansu Provincial Key Lab of Aridland Crop Science, Gansu Agricultural University, Lanzhou, 730070 China

**Keywords:** Maize (*Zea mays* L.), Mutant *lpa1*, Leaf angle, BR, WGCNA, Plant genetics, Plant hormones

## Abstract

Leaf angle, as one of the important agronomic traits of maize, can directly affect the planting density of maize, thereby affecting its yield. Here we used the *ZmLPA1* gene mutant *lpa1* to study maize leaf angle and found that the *lpa1* leaf angle changed significantly under exogenous brassinosteroid (BR) treatment compared with WT (inbred line B73). Transcriptome sequencing of WT and *lpa1* treated with different concentrations of exogenous BR showed that the differentially expressed genes were upregulated with auxin, cytokinin and brassinosteroid; Genes associated with abscisic acid are down-regulated. The differentially expressed genes in WT and *lpa1* by weighted gene co-expression network analysis (WGCNA) yielded two gene modules associated with maize leaf angle change under exogenous BR treatment. The results provide a new theory for the regulation of maize leaf angle by *lpa1* and exogenous BR.

## Introduction

Crop leaf type can significantly affect plant population structure, improve crop light energy use efficiency, and ultimately affect crop biomass and yield^1–3^. The pulvinus is an important factor in determining the formation of leaf angle, and this regulatory role has been confirmed in rice leaf^4,5^. The pulvinus size of maize was also closely related to the leaf angle, and the leaf angle of maize leafless pillow mutants *lg1* and *lg2* decreased significantly^6,7^. Research has found that plant cell elongation is regulated by hormones, which promote cell wall relaxation by stimulating the synthesis of polysaccharides necessary for flexibility and growth^8^. At the same time the elongation of cells on the proximal side of the plant's pulvinus leads to an increase in the leaf angle^9,10^.

Studies have shown that BR, as an efficient plant growth regulator, plays an important role in the development of leaf angle in monocotyledons^4,11,12^. The mutant *LC1* was obtained from the T-DNA SHIP insert population, and phenotypic analysis revealed that cell elongation in the lobe is responsible for the enlarged lobe angle. The *lc1* controls cell size at the paraxial surface of the occipital lobe through the biosynthesis and signal transduction pathway of BR, and subsequently regulates leaf angle development^13,14^. Overexpression of brassinolide upregulation-1 (*BU1*) in rice plants showed increased suture bending, increased particle size and resistance to rape octazole, an inhibitor of BR biosynthesis. The results showed that *BU1* protein is a positive regulator of BR response. It controls the bending of rice lamina joint and is a new primary response gene. It participates in two BR signaling pathways through *OsBRI1* and *RGA1*. Expression analysis showed that *BU1* was expressed in many organs such as articular plate, phloem and epithelial cells of embryos^15^. *ZmRAVL1* regulates *brd1* (brassinosteroid C-6 oxidase 1), which underlies upright plant architecture 1 (*UPA1*), altering endogenous BR content and leaf angle. The *UPA2* allele that reduces leaf angle originated from teosinte, the wild ancestor of maize, and has been lost during maize domestication. Introgressing the wild *UPA2* allele into modern hybrids and editing *ZmRAVL1* enhance high-density maize yields^16^. The maize *ZmLPA1* gene belongs to the rice loose plant structure 1 (*LPA1*) gene and the arabidopsis AtIDD15 / SHOOT GRAVITROPISM5 (*SGR5*) gene^17^. In rice *LPA1* regulates tiller angle and leaf angle by suppressing auxin hormone signalling that interacts with C-22-hydroxylated and 6-deoxo brassinosteroids^18,19^.

In this paper, we obtained the *ZmLPA1* mutant *lpa1* by mutagenesis B73 with EMS, and analyzed the leaf angle, pulvinus cell and transcriptome sequencing data of WT and *lpa1* plants, combined with WGCNA to predict the molecular mechanism of BR regulation of maize leaf angle. The results can be utilized as a theoretical basis for BR to control the leaf angle changes in maize and provide new genetic resources for maize breeding in the future.

## Materials and methods

### Plant materials and material hormone treatment

The Maize Breeding Research Group, College of Agronomy, Gansu Agricultural University provided maize WT (inbred line B73). The maize EMS mutant library of Qilu Normal University (http://elabcaas.cn/memd/) provided the source of *lpa1* (EMS3-003c97). Five maize seeds were planted in 10 cm diameter square pots containing vermiculite (10 pots each for B73 and *lpa1*), and cultivated in a light incubator at a humidity of 65% and 28 °C/24 °C for 14 h days/10 h nights with a light intensity of 6500 Lux. When maize seedlings grew to the V3 stage, they were treated with different concentrations of exogenous BR (0, 0.1, 1, 10, and 50 μmol/L) separately every 12 h, and this process was repeated 2 times. After treatment, the leaf angles of 3 maize plants were measured, and the sample material 1 cm above and below the leaf occipital area was taken and sent to Shenzhen BGI for RNA sequencing.

### Cytological analysis

After 24 h of BR treatment, 3 plants were selected for each treatment to make free-hand slices of the junction of the leaf auricle and leaf sheath on the third leaf. The experimental procedure was described by Zhang et al.^20^. Cellular structures near the maize occipital junction were observed using a LEICA DM500 microscope, an appropriate field of view was selected and photographed for each freehand section, and the size of all cells in the field of view was measured and counted using the ToupView camera system.

### Data analysis

During the data analysis, we analyzed it using Microsoft Office 2021 and IBM SPSS Statistics 27 software. The Duncan test (p < 0.05) was used to represent significant differences in WT and *lpa1* between treatments.

### Total RNA extraction and detection

Total RNA was extracted from the occipital fraction samples (18 total) of WT and *lpa1* plants treated with exogenous BR (0, 1, and 50 μmol/L) using TRIzol reagent (Invitrogen). To ensure the accuracy and completeness of the sequencing data, the total RNA concentration and quality of the extracted samples were detected by using an Agilent 21 Bioanalyzer and NanoDrop before sequencing. The total RNA of each treated sample was taken for the construction of the RNA-seq library as described by Chen et al.^21^. Then, the cDNA libraries were sequenced with the BGISEQ-500 sequencing platform, and 150 bp paired-end reads were generated. The entire process was commissioned and completed by BGI Genomics. The method used by Wang et al. was utilized for DEG detection^22^. To improve the accuracy of DEGs, genes with more than two times different times and Q-values ≤ 0.001 were defined and screened as significantly differentially expressed genes. Conduct in-depth cluster analysis and functional enrichment analysis of genes that are differentially expressed. The original sequencing reads have been submitted to the SRA at NCBI (Accession number: PRJNA1013802).

### Weighted gene co-expression network analysis

WGCNA is the main method to construct a gene co-expression network. In this paper, the expression data of 18 samples from WT and *lpa1* treated with different concentrations of exogenous BR were analyzed by R packet WGCNA^23^. The threshold was set as follows: FPKM = 1, fold threshold = 1.5, and the minimum number of genes in the module was set to 30. Furthermore, the correlation between module eigenvalues and phenotypic trait data was determined through module correlation analysis. Pearson correlation was used to calculate the correlation coefficient between phenotypic trait data and gene module eigenvalues, and an analysis of the correlation heatmap was performed. The weight network mapping tool of the OmicShare cloud platform was used to draw the network visualization map of the first 150 genes in the module.

### Ethical statement

The authors statement that the experimental research and field studies of plants (cultivated or wild), including the collection of plant materials, are in accordance with the relevant institutional, national and international guidelines and legislation. All methods concerning plants and plant materials were operated in accordance with the relevant guidelines in the “Materials and methods” section.

## Results

### Screening of the optimal concentration of exogenous BR

**Figure 1:**
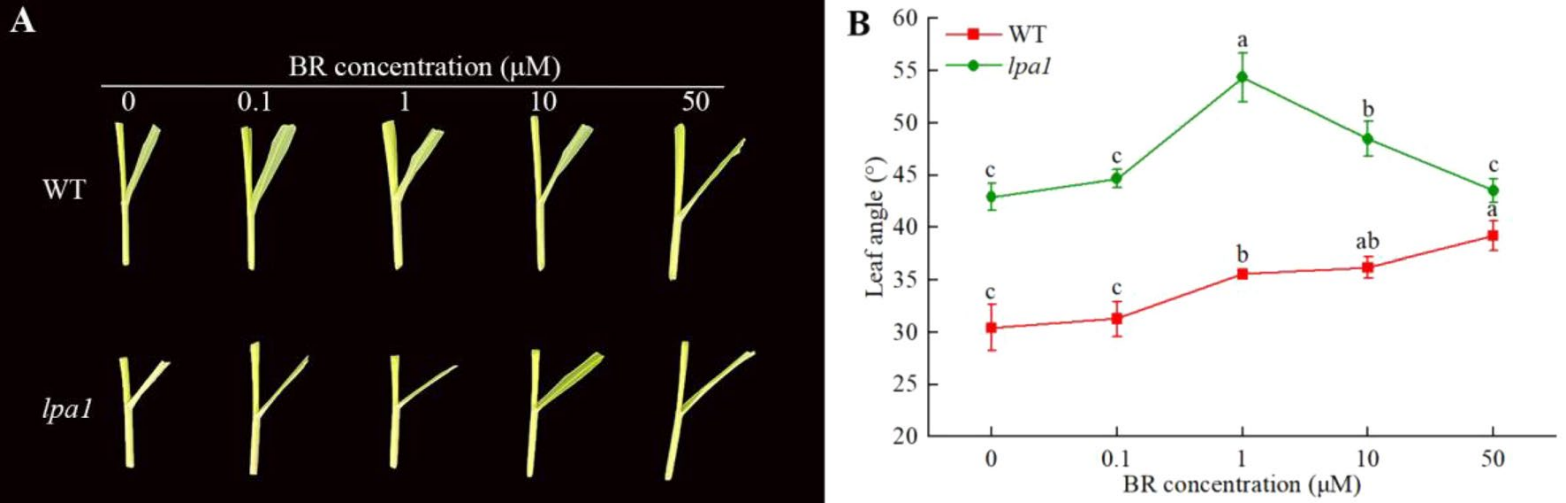
Exogenous BR has an effect on the leaf angle of WT and *lpa1*. (**A**) Morphology of maize leaf angle under BR treatment; (**B**) size of maize leaf angle under BR treatment. Note: Letters represent a significant difference at the 0.05 level, the same as below.

The results showed that the change in leaf angle in plants was closely related to plant hormones, especially BR, which played an important role in the formation of leaf angle. Therefore, we performed BR response analysis experiments on WT and lpa1, and treated maize materials at the V3 stage with different concentrations of hormones to analyze the effects of different concentrations of BR on leaf angle. The results showed that with increaseing of exogenous BR concentration, the leaf angles of WT and lpa1 increased. When the concentration of BR increased to 1 μmol/L, the leaf angle of WT continued to increase, while the leaf angle of *lpa1* gradually decreased, indicating that *lpa1* was less sensitive to BR. At 1 μmol/L BR treatment, the WT leaf angle was 35.6°, mutant *lpa1* and a peak of 54.3°, with a difference from WT of 18.7°. The results showed that 1 μmol/L BR was the optimal concentration to adjust the leaf angle of *lpa1* (Fig. 1).

### Cytological observations of Ligular region

**Figure 2:**
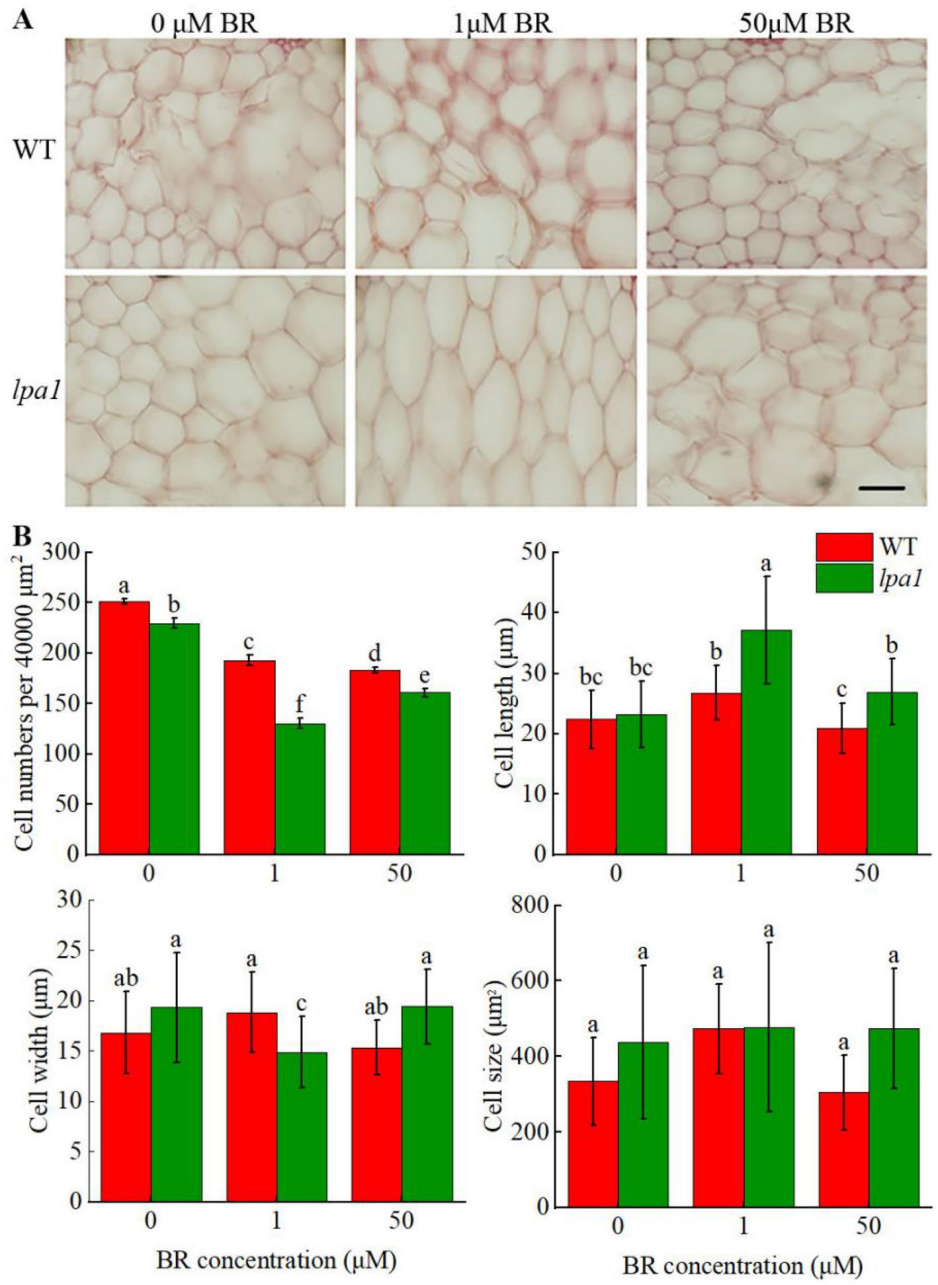
Cytological analysis of leaves near the occipital lobe of WT and *lpa1* treated with exogenous BR. (**A**) Leaf cell structure of close to the pulvinus of two materials treated with exogenous BR. Bars = 20 μm; (**B**) cell number, length, width and size of leaf cells of WT and *lpa1* treated with exogenous BR close to the occipital lobe.

To study the effect of exogenous BR on maize leaf angle, we made cytological observations on the cellular structure of WT and *lpa1* at the inferior auricle in the V3 stage after treatment with BR. The results showed differences in the number of cells per unit area, cell length, cell width, and cell size between maize materials treated with different concentrations of BR. We observed that the most obvious changes in the two materials under 1 µmol/L BR treatment, in which the average length of WT cells was 26.78 µm, which increased 19.35% compared with the control group, and the average length of *lpa1* cells was 37.08 µm, which increased 59.48% compared with the control group; The average cell size for WT was 472.82 µm^2^, an increase of 41.71% from control, and the average cell size for *lpa1* was 477.07 µm^2^, up 9.16% from control (Fig. 2). Our study found that the number of *lpa1* cells treated with the same field of view was significantly less than that of WT, and the increase in leaf angle between WT and *lpa1* under exogenous BR treatment was inversely proportional to the number of cells.

### DEGs analysis in WT and *lpa1* at different BR concentrations

**Figure 3:**
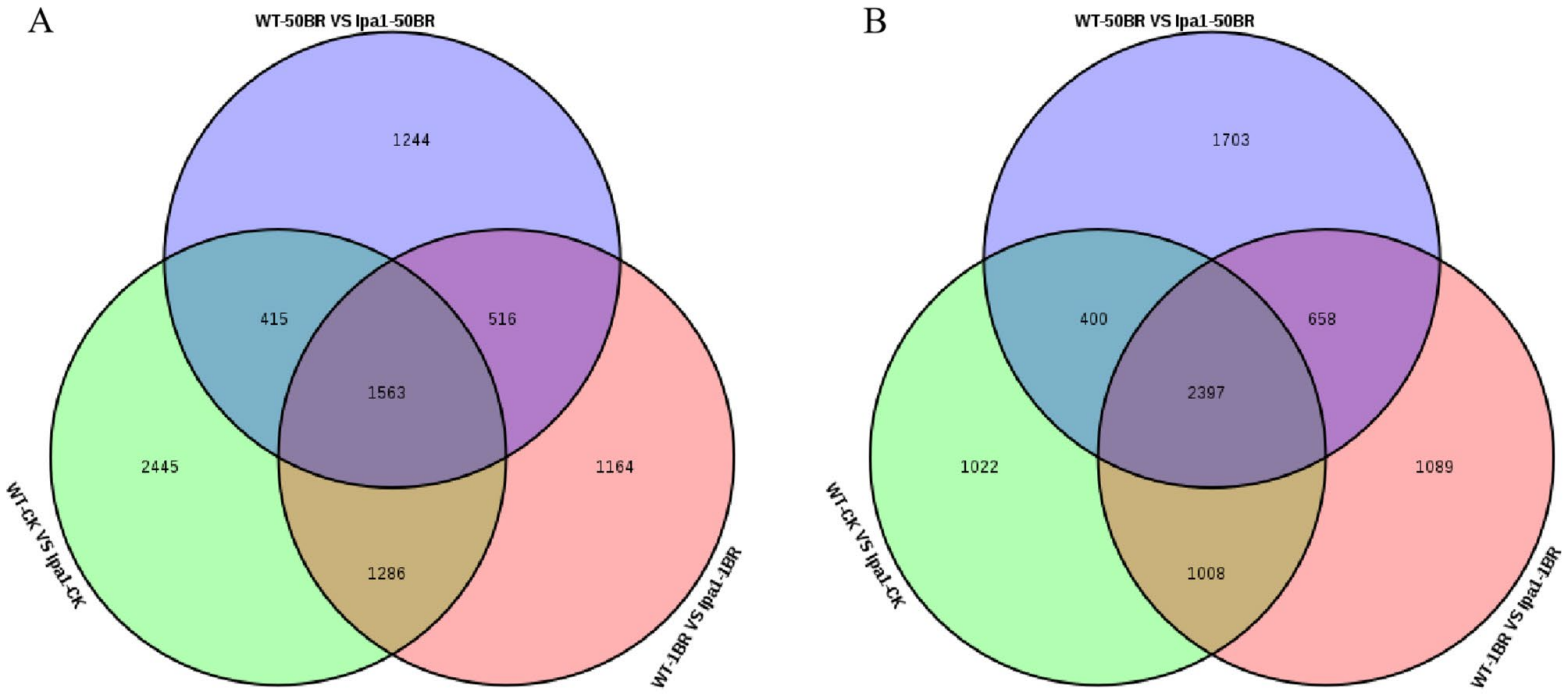
Analysis of DEGs in WT and *lpa1* under different concentrations of BR treatment. (**A**) Upregulation of DEGs in WT and *lpa1* under BR treatment; (**B**) downregulation of DEGs in WT and *lpa1* under BR treatment.

Using FPKM as a measure of gene expression, a total of 15809 DEGs were identified for WT and *lpa1* at different concentrations of BR treatment (Fig. 3). 10536 DEGs (5709 upregulated, 4827 downregulated) in the WT-CK VS *lpa1*-CK comparison group (Supplementary Table S1); 9681 DEGs (4529 up and 5152 downregulated) in the WT-1BR VS *lpa1*-1 BR comparison group (Supplementary Table S2); and 8896 DEGs (3738 upregulated, 5158 downregulated) in the WT-50BR VS *lpa1*-5 0 BR comparison group (Supplementary Table S3). A total of 3965 DEGs were identified in WT and *lpa1* under different concentrations of BR treatment, of which 1563 were up-regulated and 2398 were down-regulated (Fig. 3A, B). From the above analysis, we found that WT and *lpa1* have very many DEGs under three different treatments, so it is speculated that the mechanism of the two responses to exogenous BR may be different.

### GO classification analysis of WT and *lpa1* DEGs at different BR concentrations

**Figure 4:**
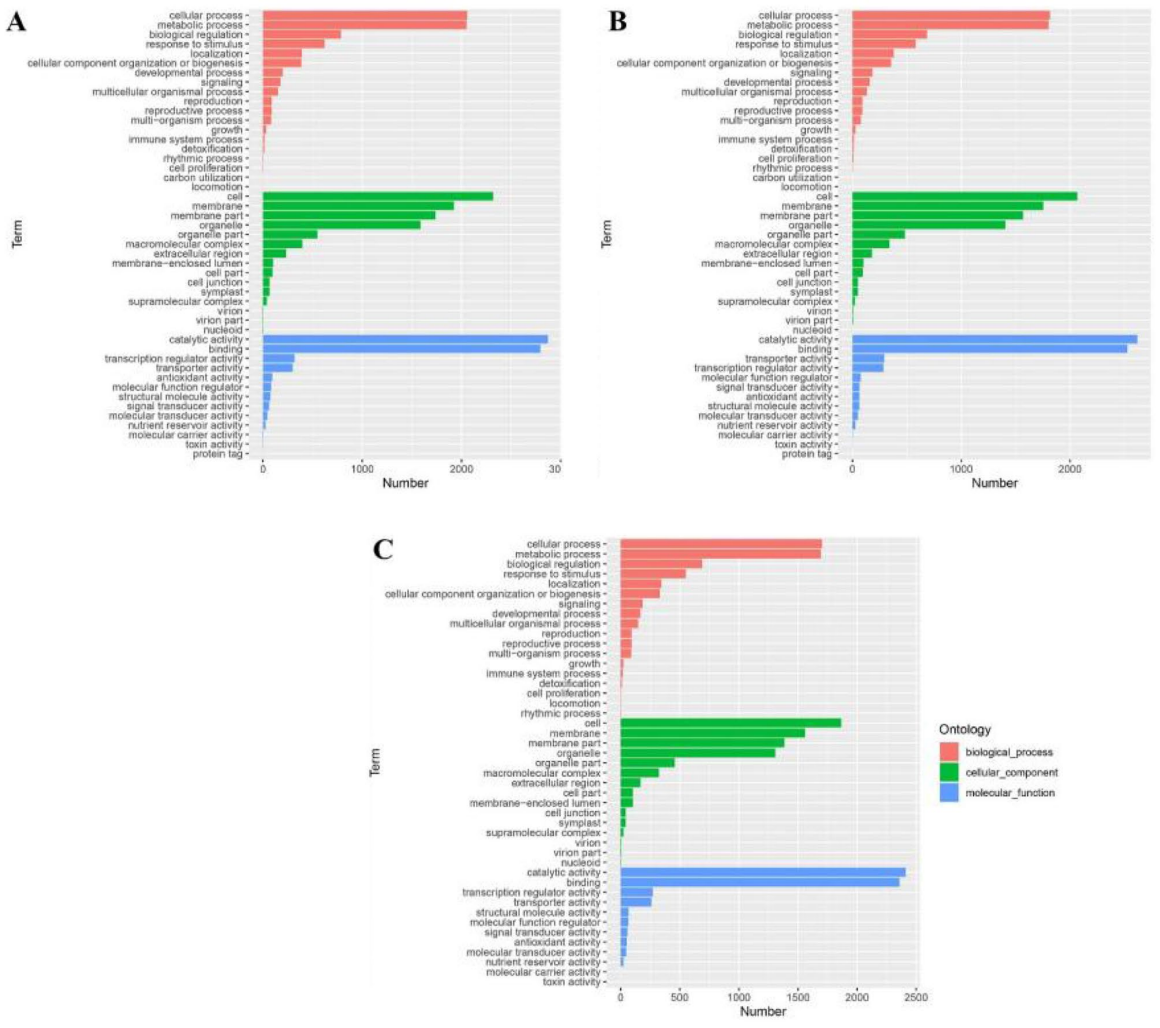
GO annotation of DEGs in WT and *lpa1* identified under different concentrations of exogenous BR treatment. (**A**) WT-CK VS *lpa1*-CK; (**B**) WT-1BR VS *lpa1*-1BR; (**C**) WT-50BR VS *lpa1*-50BR. CK = control; 1BR = 1 μmol/L BR and 50BR = 50 μmol/L BR.

To better understand the biological functions of WT and *lpa1* under different concentrations of exogenous BR treatment, we performed gene ontology (GO) classification, and the main biological processes enriched for DEGs in the three comparison groups (WT-CK VS *lpa1*-CK, WT-1BR VS *lpa1*-1 BR and WT-50BR VS *lpa1*-50 BR) were biological processes (BP), cellular components (CC) and molecular functions (MF) (Fig. 4). The DEGs between material WT-CK and *lpa1*-CK were mainly enriched in BP including drug catabolic process and cell wall organization or biogenesis, CC included extracellular region and anchored component of plasma membrane, MF included tetrapyrrole binding and hydrolase activity, hydrolyzing O-glycosyl compounds; the DEGs between material WT-1BR and *lpa1*-1 BR were mainly enriched in BP include cell wall organization or biogenesis and carbohydrate metabolic process, CC included intrinsic component of membrane and integral component of membrane, MF included catalytic activity and hydrolase activity, acting on glycosyl bonds; The DEGs between material WT-50BR and *lpa1*-50 BR were mainly enriched in BP including response to auxin and cell recognition, CC included extracellular region and intrinsic component of membrane, and MF included monooxygenase activity and oxidoreductase activity.

### Pathway enrichment analysis of WT and *lpa1* DEGs under different BR concentration*s*

**Figure 5:**
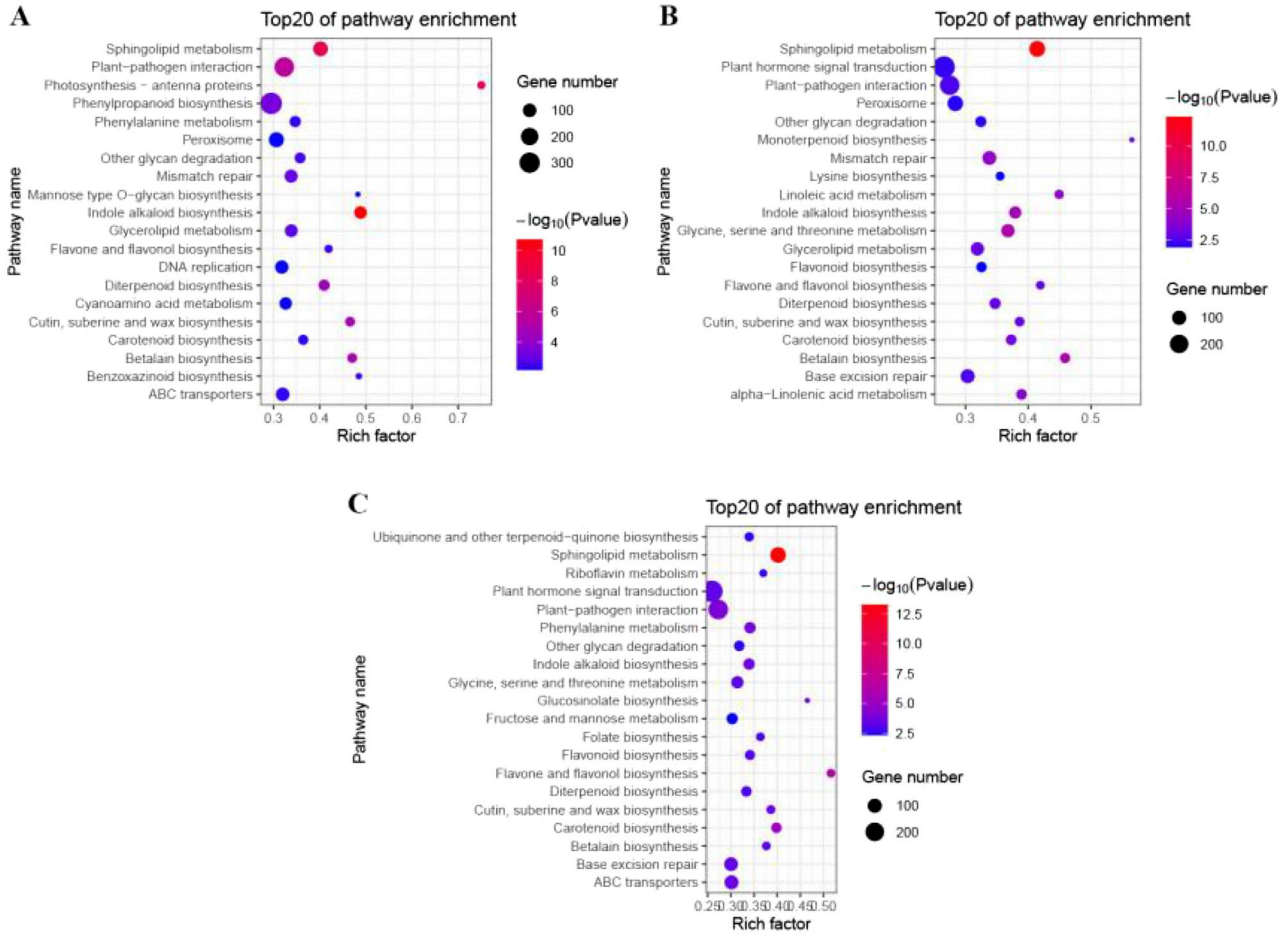
Pathway enrichment analysis of WT and *lpa1* exposed to different treatments. (**A**) WT-CK VS *lpa1*-CK; (**B**) WT-1BR VS *lpa1*-1BR; (**C**) WT-50BR VS *lpa1*-50BR. CK = control; 1BR = 1 μmol/L BR and 50BR = 50 μmol/L BR.

Under the same treatment for WT and *lpa1*, the three comparison groups are WT-CK VS *lpa1*-CK, WT-1BR VS *lpa1*-1BR, and WT-50BR VS *lpa1*-50BR, respectively. The three comparison groups have 9 common metabolic pathways, namely indole alkaloid biosynthesis, sphingolipid metabolism, plant-pathogen interaction, cutin, suberine and wax biosynthesis, betalain biosynthesis, other glycan degradation, flavone and flavonol biosynthesis, carotenoid biosynthesis. In the comparison group, WT-CK VS *lpa1*-CK had six distinct metabolic pathways, which are photosynthesis–antenna proteins, phenylpropanoid biosynthesis, benzoxazinoid biosynthesis, cyanoamino acid metabolism, mannose type O-glycan biosynthesis and DNA replication; WT-1BR VS *lpa1*-1BR had four distinct metabolic pathways, which are linoleic acid metabolism, alpha-Linolenic acid metabolism, monoterpenoid biosynthesis and lysine biosynthesis; WT-50B VS *lpa1*-50B had five distinct metabolic pathways, which are glucosinolate biosynthesis, folate biosynthesis, ubiquinone and other terpenoid-quinone biosynthesis, riboflavin metabolism and fructose and mannose metabolism (Fig. 5).

### Transcription factor family analysis

**Figure 6:**
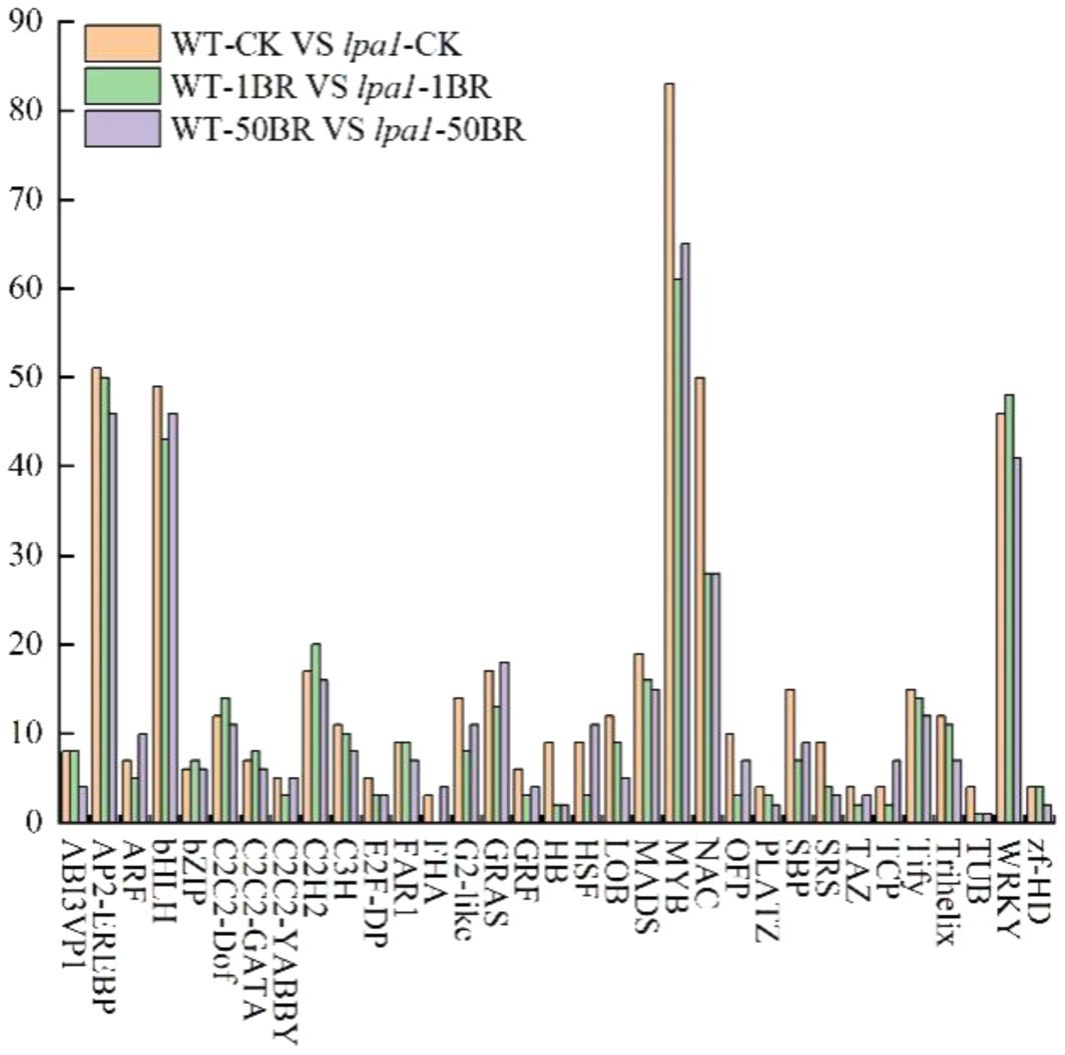
Transcription factor analysis of differential expression in WT and *lpa1* under exogenous BR treatment.

We predicted DEGs with the ability to encode transcription factors and classified and counted the families of transcription factors to which these genes belonged. RNA-seq results showed that many TFs were differentially regulated under BR treatment. Here, we only show TF families with 3 or more DEGs (Fig. 6). We found that 33 TFs were regulated differently under exogenous BR treatment in WT and *lap1*. The top five TF families of DEGs were MYB, AP2-EREBP, bHLH, WRKY and NAC. Under 1 μmol/L BR treatment, the TF families with obvious differences were MYB, NAC, WRKY, SBP, HB and OPF. We identified significantly more TF DEGs when compared with WT and *lap1* at different concentrations of BR treatment. These DEGs belong to the same TF family, indicating that *lap1* could alter the expression of these TF genes in response to exogenous BR treatment. These DEGs may be the key genes involved in the maize leaf angle response to exogenous BR.

### Gene co-expression network analysis

**Figure 7:**
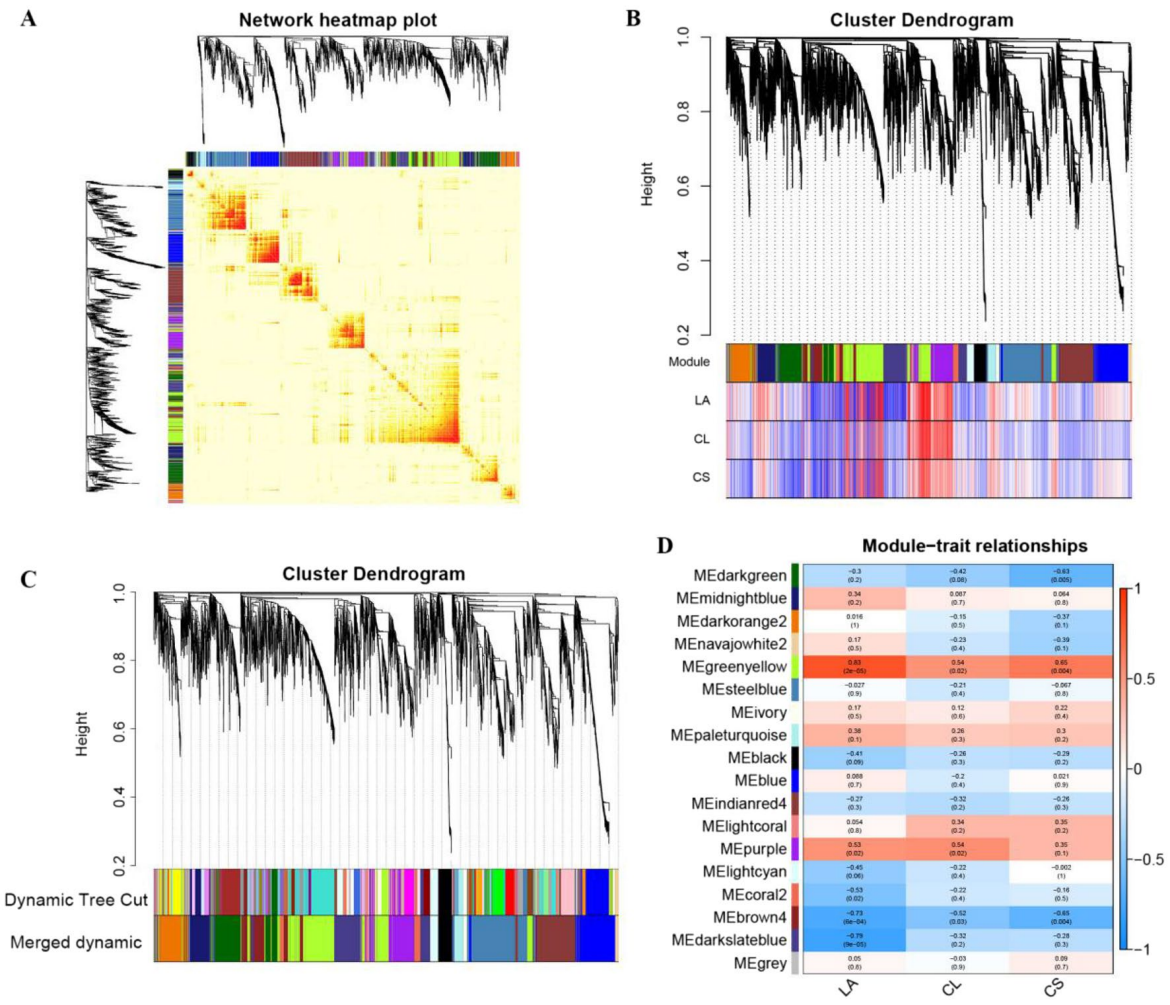
Sample clustering and gene module correlation analysis. (**A**) Correlation heatmap between modules; (**B**) correlation between gene modules and phenotypes; (**C**) hierarchical cluster analysis of co-expression genes; (**D**) heatmap of the correlation between modules and sample types (grey modules are unassigned gene module). Notes: LA, Leaf angle; CL, Cell length; CS, Cell size.

WGCNA is a commonly used method for constructing gene co-expression networks that can identify gene sets with similar expression patterns (i.e. module modules). We performed transcriptome sequencing of 18 samples (WT and *lpa1*, 0, 1 and 50 μmol/L BR treatment and 3 biological replicates), filtered out genes with mean FPKM expression below 1, had a total of 37990 genes, and set the similarity threshold of Fold > 0.5 for Module fusion to build a co-expression network module. Based on the correlation between module characteristic genes and phenotypic traits of maize LA (leaf angle), CL (cell length) and CS (cell size), the modules significantly related to phenotype were screened. A total of 18 modules meeting the conditions were screened, of which the MEgreenyellow and MEpurple modules module had the highest correlation with the leaf angle of maize. The results showed that the maize leaf angle was significantly positively correlated with the MEgreenyellow and MEpurple modules, and the correlation coefficients were 0.83 and 0.53 respectively (Fig. 7). We conducted KEGG enrichment analysis on significantly correlated genes in these two modules, and found that the genes in the MEgreenyellow module mainly participate in Plant hormone signal transduction, Carbon metabolism, Glycolysis/Gluconeogenesis; The genes in the MEpurple module mainly participate in Cysteine and methionine metabolism, Carbon metabolism, Plant hormone signal transmission, which may play a role in regulating leaf angle in maize (Supplementary Tables S4, S5).

### Analysis of hub genes interaction network in the module

**Figure 8:**
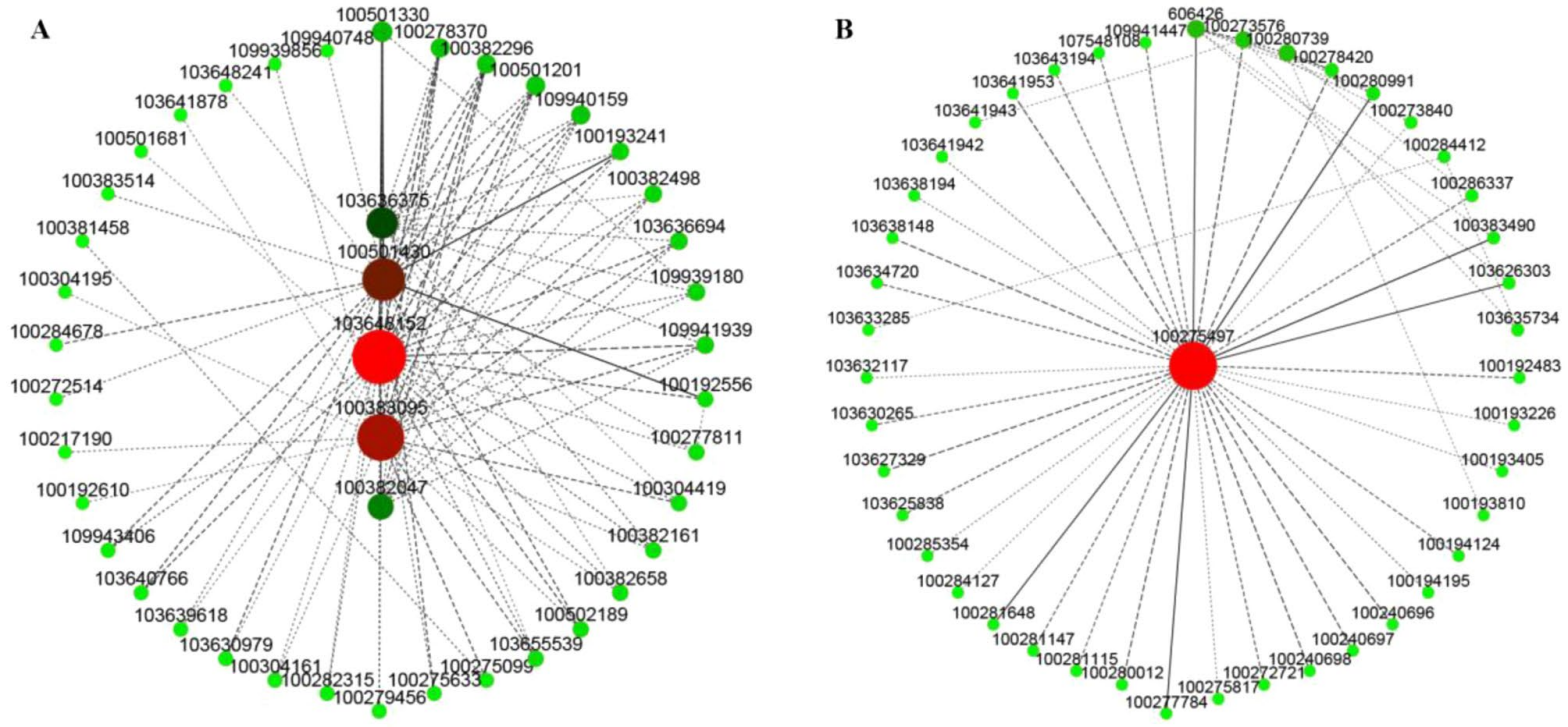
Analyzing the interactions between core gene networks within the co-expression module. (**A**) Interaction analysis of core genes in the MEgreenyellow module; (**B**) interaction analysis of core genes in the MEpurple module.

In this study, we performed gene network visualization and gene connectivity analysis for the MEgreenyellow and MEpurple module genes. To mine the Hub genes related to the angle of maize leaves, we utilized the hub genes and their interacting genes to map the gene co-expression network (Fig. 8). Among the five central genes in the green yellow (0.83) module, 103648152, 100383095 and 100382047 are three genes of unknown function, 100501430 was associated with O-fucosyltransferase family protein, and 103636375 was associated with G-rich sequence factor 1 (Fig. 8A). In the purple (0.53) module, the function of central gene 100275497 is unknown (Fig. 8B). We speculate that these six genes were involved in maize leaf angle regulation in the process of exogenous BR increasing the *lpa1* leaf angle.

## Discussion

### Application of mutants in plant leaf angle

Currently, many plant gene defective mutants are used in gene research, and the use of mutants can reveal the function of genes in plants^24–27^. Identification of wheat mutants with upright leaf phenotype caused by lamina joint developmental defects, the results of the gene encoding squamous promoter binding-like (SPL) protein deletion *TaSPL8*, *TaSPL8* knockout mutant in the number of ears due to the absence of lamina joints, the structure of the compact, especially at high planting density^27^. Rice leaf inclination 2 (*lc2*) mutant compared with wild-type plants found that *LC2* was mainly expressed in lamellar joints during leaf development, especially under the phytohormones ABA, GA, IAA, and BR induced, and *LC2* defective resulted in altered expression of cell division and hormone-responsive genes, suggesting that LC2 plays an important role in regulating leaf tilt and hormonal effects^14^. Using the rice functional mutant slender grain dominant (*slg-D*), the narrower the mutant grain, the larger the leaf angle, the study showed that the *slg-D* phenotype is caused by the increased expression of BAHD acyltransferase-like protein gene *SLG*^28^. The maize mutant library also provides a good resource for studying maize plant types and physiological traits^29^. In the early stage of the experiment, we used the obtained mutant *lpa1* with an enlarged leaf angle, and found that the *lpa1* leaf angle was greater than B73, the leaf length became shorter, and the leaf width narrowed^19^.

### Effect of BR on leaf angle of WT and *lpa1*

The development of the plant leaf angle is affected by many factors, and hormones play an important regulatory role in the development of the leaf angle^30,31^. Therefore, the role of BR in the process of plant leaf angle development and growth is not negligible, and we found that there were 6 DEGs associated with the brassinosteroid biosynthesis pathway by KEGG analysis of DEGs of WT and *lpa1*, and one of the genes encoding steroid 5α-reductase found that the oxidative stress resistance of arabidopsis DET2-deficient plants was enhanced, which may be due to physiological stress caused by long-term lack of BRs in plants. This in turn activates the structural expression of some defense genes, and then activates the activity of related enzymes^32^. Five genes belonging to CYP90B1 (cytochrome P450 90B1), and the catalytic activity and substrate specificity of CYP90B1 in Arabidopsis thaliana confirmed that brassinol was the preferred substrate for CYP90B1, and CYP90B1 catalyzed 325 times that of brassinol, and is a key enzyme in regulating the natural abundance of brassinolides^33^. One gene encoding the brassinosteroid LRR receptor kinase BRI1 was also discovered, a receptor-like kinase with a special structure, complex leucine-containing repeats, which was found to be a receptor protein for BR and is essential in the BR signaling pathway^34^. Therefore, we can speculate that the increase in the *lpa1* leaf angle may be caused by *ZmLPA1* gene mutation, which affects the biosynthesis of brassinolactone and the BRI1 receptor protein in *lpa1*.

### Effects of different signal transduction pathways on maize leaf angle

In this study, six genes associated with maize leaf clip angle were selected by |log2FC|> 1 and P < 0.001. We found that one extenxin B7 gene (Expansin-B7, gi |541908|) was downregulated under exogenous 1 μmol/L BR treatment. The high expression level of extenxin promoted cell growth and elongation, while the expression of this gene was downregulated, indicating that it inhibited plant cell elongation and enhanced cell division ability. The expression of one cysteinase gene (Xylem cysteine proteinase 2, gi |100283748|) was downregulated under exogenous 1 μmol/L BR treatment, which mainly plays an important role in vascular bundle organization in maize, and may be related to leaf angle. Studies have shown that the overexpression of the GTP binding protein gene OsRab7B3 promoted the transgenic rice leaf senescence^35^. This study found that a Rop 3 small GTP binding protein gene (Rop3 small GTP binding protein, gi |541774|) under exogenous 1 μmol/L BR treatment expression was downregulated, indicating that BR through the inhibition of GTP binding protein gene expression and inhibition of maize leaf senescence, enhanced the cell division ability, thus increase the leaf angle. Finding 1 BZIP transcription factor protein gene (BZIP transcription factor, gi |103641983|) downregulated expression under exogenous 1 μmol/L BR treatment indicates that the appropriate concentration of BR inhibited the expression of this gene blocking light signaling, bending the plant organs and possibly leading to enlarged leaf angle. Two genes were related to leaf growth and development, and one galactosyn 11 gene (Fasciclin-like arabinogalactan protein 11, gi |103643069|) were downregulated under exogenous 1 μmol/L and 50 μmol/L BR treatment, which mainly regulated cell wall integrity, root growth and stem development in arabidopsis, which may be related to leaf angle. Under exogenous 50 μmol/L BR treatment, a putative MATE family transporter gene (putative MATE efflux family protein, gi |100501847|) was downregulated. It is speculated that a high concentration of BR inhibited the expression of MATE family transporter genes and promoted maize mesotyl cell elongation, which may affect the size of the maize leaf angle.

### DEGs analysis of WT and *lpa1* under exogenous BR treatment

**Figure 9:**
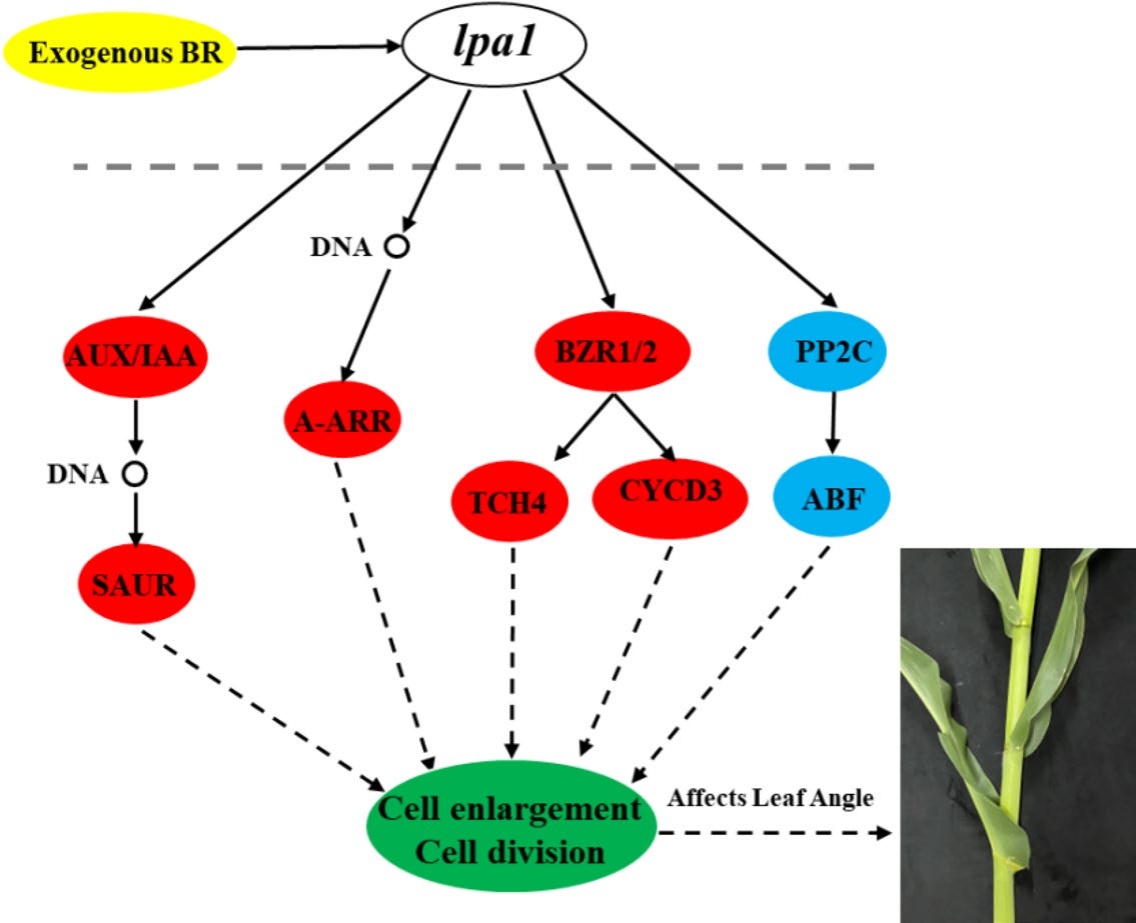
Molecular model of exogenous BR regulating leaf angle in maize (Red indicates a higher expression level of related genes in *lpa1* compared to WT, while blue indicates a lower expression level of related genes in *lpa1* compared to WT).

Many studies have shown that plant transcriptome sequencing analysis has an important role in the process of mining functional genes associated with plant phenotypes^36,37^. By analyzing the phytohormone signal transduction pathways for the DEGs of WT and *lpa1,* we found that auxin-related genes AUX/IAA and SAUR; cytokinin-related gene A-ARR; and brassinosteroid-related genes BZR1/2, TCH4 and CYCD3 were significantly up-regulated, while abscisic acid-related genes PP2C and ABF were significantly down-regulated (Supplementary Table S6). Auxin response factor (ARF) is a type of auxin response element in downstream auxin response genes after receiving signals from upstream. It has been found that ARF in plants is likely used by selecting target genes as transcription factors to bind to auxin response elements in the promoter of auxin-regulated genes, which activate or inhibit the transcription of these genes based on the specific domains of the protein^38^. As the largest auxin early response genes, most of the small auxin-up RNA (SAUR) expression is regulated by auxin, and the diversity and specificity of the transcriptional level not only gives the cellular level has the same function of SAUR regulation of different plant growth and development process abilities but also shows that SAUR19-24 can be used as a positive effector of plant cell expansion^39^. A-ARRs are negatively regulated by autophagosomes (EXO70D) in a phosphorylation-dependent manner. In the absence of cytokinin, unphosphorylated A-ARRs are ubiquitinated and shuttle to the 26S proteasome for degradation, and thus their stability is thus regulated by cytokinin and proteasomal degradation pathways^40,41^. Brassinazole resistance factor (BZR) is a key signaling element in the plant hormone brassinotone signal transduction pathway, which regulates plant growth and environmental response by activating the expression of related genes^42^. TCH4 (TOUCH4), also known as XTH 22, is a member of the xyloglucan endotransglucosylase/hydrolase (XTH) family member that encodes an XET enzyme. This enzyme is able to transfer xyloglucan from the cell wall in plant morphogenesis, thus affecting cell wall formation and degradation^43^. Both ABA-insensitive 1 (ABI1) and ABI2 genes encode PP2C protein phosphatase in Arabidopsis thaliana, which can negatively regulate ABA signal transduction pathway (The ABI1 and ABI2 protein phosphatases 2C act in a negative feedback regulatory loop of the abscisic acid signalling pathway). PP2C can regulate the plant ABA response by regulating the activities of the SNF1-related protein kinase 2 (SnRK2) family SnRK2.2/SRK2D, SnRK2.3/SRK2I and SnRK2.6/SRK2E/OST1 kinases^44,45^. The results show that under the influence of exogenous BR, *lpa1* affects the regulation of the four plant hormones, IAA, CTK, BR and ABA. Combined results of increased leaf angle, increased cell volume in the pulvinus region and DEGs analysed in maize WT and *lpa1* under exogenous BR treatment, we established a molecular model of the response mechanism of the leaf angle of *lpa1* to exogenous BR (Fig. 9).

## Conclusions

In this study, we used the obtained mutant *lpa1* with increased corn leaf angle to explore the regulatory mechanism of exogenous BR on *lpa1* leaf angle. WT and *lpa1* were treated with different concentrations of BR, the optimal BR concentration was 1 μmol/L, and transcriptome sequencing analysis was performed. The results show that BR regulating the increase of *lpa1* leaf angle is mainly related to the genes of cell elongation and cell division, which is consistent with our cytology observations. Using 15,809 genes to construct a weighted gene co-expression network, we obtained modules MEgreenyellow (0.83) and MEpurple (0.53) that were significantly positively correlated with phenotypes. They were significantly positively correlated with changes in the angle between maize leaves. By constructing a module gene interaction network diagram, we obtained 6 candidate genes that could participate in the regulation of maize leaf angle.

### Supplementary Information

Supplementary Tables.

## Data Availability

Data supporting the findings of this work are available within the paper and its Supplementary Table files. The RNA-seq data generated in this study have been deposited in the National Center for Biotechnology Information Sequence Read Archive database under accession PRJNA1013802 and PRJNA851970 (*lpa1* related RNA-seq data data were uploaded to the PRJNA1013802, B73 related RNA-seq data data were uploaded to the PRJNA851970).
